# Genetic selection against intrauterine growth retardation in piglets: a problem at the piglet level with a solution at the sow level

**DOI:** 10.1186/s12711-018-0417-7

**Published:** 2018-09-18

**Authors:** Stephanie M. Matheson, Grant A. Walling, Sandra A. Edwards

**Affiliations:** 1 Agriculture, School of Natural and Environmental Sciences, University of Newcastle, Newcastle upon Tyne, NE1 7RU UK; 2 JSR Genetics, Southburn, Driffield, East Yorkshire, YO25 9ED UK

## Abstract

**Background:**

In polytocous livestock species, litter size and offspring weight act antagonistically; in modern pig breeds, selection for increased litter size has resulted in lower mean birth weights, an increased number of small piglets and an increased number of those affected by varying degrees of intrauterine growth retardation (IUGR). IUGR poses life-long challenges, both mental, with morphological brain changes and altered cognition, and physical, such as immaturity of organs, reduced colostrum intake and weight gain. In pigs, head morphology of newborn piglets is a good phenotypic marker for identifying such compromised piglets. Growth retardation could be considered as a property of the dam, in part due to either uterine capacity or insufficiency. A novel approach to this issue is to consider the proportion of IUGR-affected piglets in a litter as an indirect measure of uterine capacity. However, uterine capacity or sufficiency cannot be equated solely to litter size and thus is a trait difficult to measure on farm.

**Results:**

A total of 21,159 Landrace × Large White or Landrace × White Duroc piglets (born over 52 weeks) with recorded head morphology and birth weights were followed from birth until death or weaning. At the piglet level, the estimated heritability for IUGR (as defined by head morphology) was low at 0.01 ± 0.01. Piglet direct genetic effects of birth weight (h^2^ = 0.07 ± 0.02) were strongly negatively correlated with head morphology (− 0.93), in that IUGR-affected piglets tended to have lower birth weights. At the sow level, analysis of the proportion of IUGR-affected piglets in a litter gave a heritability of 0.20 ± 0.06, with high and negative genetic correlations of the proportion of IUGR-affected piglets with average offspring birth weight (− 0.90) and with the proportion of piglets surviving until 24 h (− 0.80).

**Conclusions:**

This suggests that the proportion of IUGR-affected piglets in a litter is a suitable indirect measure of uterine capacity for inclusion in breeding programmes that aim at reducing IUGR in piglets and improving piglet survival.

## Background

The antagonistic life-history trade-off between litter size and offspring size [1–4] originates from the allocation of limited maternal reserves, i.e. energy, nutrients, and abdominal space available for offspring [5]. In polytocous livestock species, litter size is studied in order to increase prolificacy, which is the result of complex interactions between male, female, and embryo genotypes [6], although genetic or permanent environmental factors are of low importance due to the high variability in litter size [7]. The success of selection for high prolificacy may be due largely to an increase in the number of corpora lutea [8, 9], with associated negative consequences for the resulting foetal-placental units [9–11]. Indeed, in pigs, it has been shown that selection for increased litter size leads to a significantly lower mean birth weight and a greater percentage of small piglets born [12, 13], with the possibility of various degrees of intrauterine growth retardation occurring in these small piglets [14].

In pigs, intrauterine growth retardation (IUGR) has been associated with impaired foetal and placental growth [15], which can result in lower birth weights and a higher brain to liver weight ratio [16] due to the ‘brain-sparing effect’. This is part of a foetal adaptive reaction to placental or nutritional insufficiency [17], which may have permanent effects on the structure, physiology and metabolism of the body [18, 19], intestinal morphology and enzyme secretion [20–22]. Wang et al. [23] showed that proteins related to energy supply, protein metabolism and muscle structure, function and proliferation are differentially expressed in IUGR-affected piglets, which indicates impaired metabolism and reduced growth and development of muscle. Thus, IUGR poses economic problems for subsequent commercial meat production, such as reduced feed conversion efficiency and a decreased percentage of meat [24] and increased percentage of body fat in the carcass [25].

One suggested solution to reduce the incidence of IUGR-affected piglets due to gestational-undernutrition is to provide the sow with a nutritional intervention during pregnancy [26, 27]. However, some studies have found that increasing global maternal nutrition has no effect on piglet performance and muscle traits [28], while other studies observed that both maternal under-nutrition and over-nutrition may stunt foetal growth [29, 30].

An alternative approach to reduce IUGR is to select against the IUGR phenotype, either directly at the piglet level or at the maternal level. Some easily classified fitness traits at the offspring level are heritable and suitable for direct selection, i.e. offspring vitality or vigour [31, 32]. In pigs, as in other polytocous species (e.g. rats [33]), IUGR-affected offspring display a phenotype that is easy to categorise, i.e. an altered head morphology [34, 35] with three characteristics: (1) a steep, dolphin-like forehead, (2) bulging eyes, and (3) wrinkles perpendicular to the mouth.

Although the foetal genome is an important factor for growth potential in utero, there is evidence that suggests that the intrauterine environment is a major determinant of foetal growth [30]. In this case, if selection at the piglet level is not effective, selection at the maternal level could be based on the proportion of IUGR-affected piglets in a litter. Bazer et al. [36, 37] showed that greater embryonic loss, which is associated with a larger number of embryos in the uterus, is due to maternal limitations and not to limitations of the embryo. The proportion of IUGR-affected piglets in a litter may depend, in part, on the available uterine capacity, which can be defined as the ability of the uterus to maintain the appropriate development of some number of conceptuses [38]. However, uterine capacity, as defined by Webel and Dzuik [39], is difficult to measure directly on farm but the proportion of IUGR-affected piglets in a litter could be an indirect measure that could be used for selection.

The amount of genetic variation in head morphology of IUGR-affected piglets at the piglet and sow levels can be compared by estimating either the narrow-sense heritability, *h*^2^, i.e. the standardised additive genetic variance, *V*_*g*_, or the evolvability, *I*_*A*_, i.e. the square of the coefficient of the additive genetic variance, *I*_*A*_ = 100 × *V*_*g*_/*mean*^2^ [40]. The evolvability of a trait refers to the ability of a population to respond to a potential selective challenge [41], i.e. its ability to generate adaptive genetic diversity, and is equal to the “expected percent change in a trait under a unit strength of selection” [42]. Therefore, the evolvability of a trait refers to the capacity of a population to generate heritable and selectable phenotypic variation [43], which suggests that a greater evolvability allows for greater selection possibilities. *I*_*A*_ is a mean-scaled and dimensionless statistic, like narrow-sense heritability, *h*^2^, and, thus, is also suitable for the comparison of traits at both the maternal and offspring level.

Thus, the objective of this study was to determine if the incidence of immature piglets could be reduced by: (1) selection at the piglet level by recording IUGR head morphology of IUGR-affected piglets used as an indirect measure for IUGR; or (2) selection at the sow level based on the proportion of IUGR-affected piglets born in a litter, as a candidate for indirect measurement of uterine insufficiency.

## Methods

This study was conducted in accordance with the Newcastle University Ethics Policy for Research, Teaching and Consultancy. All pigs were maintained in accordance with the Welfare of Farmed Animals (England) Regulations 2007.

### Animals

Data were collected for 52 weeks in 2015 and 2016 on 21,159 piglets from 1575 litters (866 individual sows) at a commercial multiplier sow herd located in the UK, which produces crossbred gilts. The unit consists of a sow herd of approximately 750 Landrace sows, which are inseminated artificially with either White Duroc or Large White semen. At insemination, animals in standing oestrus were served up to 3 times at 12- to 14-h intervals with a semen dose of 2.7 billion spermatozoids. The decision of whether to serve an animal for a third time depended on whether it still demonstrated standing oestrus.

During gestation, animals were fed on a standard gestation curve according to the season. In the summer months (April–September), gilts were given 2.2 kg sow meal post-service and 2.5 kg prior to farrowing, whereas sows were given 2.5 kg sow meal at service and 3.0 kg sow meal at farrowing. During the winter months (October–March), gilts were given 2.5 kg sow meal at service and 3.0 kg sow meal at farrowing, with sows receiving an additional 0.5 kg compared to the gilts. Each ration contained crude protein at 10.50%, oils/fats at 5.25%, crude fibre at 4.25%, crude ash at 4.75%, and digestible lysine at 0.43%. The net energy of the ration was 9.35 MJ/kg.

### Animal husbandry

After insemination, sows were group-housed in gestation pens and remained in their gestation pen cohorts until shortly before farrowing, except for those removed because of lameness or aggression issues. During farrowing (and until weaning), sows and piglets were maintained in standard farrowing crates (0.62 × 2.40 m), with a heated piglet area (heated mat or heat lamp) to the front or side of the sow. In general, no assistance was required during farrowing, except in the case of prolonged birth, and all piglets remained in their birth litters until processing at 18–24 h after farrowing, when piglets were first handled for tail docking, teeth grinding and ear tagging for identification of females (males were not identified). All piglets were then cross-fostered to equalize the number and size of piglets in a litter and maximise chances of survival by mixing all piglets born on the same day and then allocating them by size to the available sows.

### Data collection

For sows, data collection included parity, gestation length (GL), total litter size (LITTER SIZE i.e. the number of full-term piglets born alive + born dead, but not including mummified piglets), and the numbers of piglets born alive, born dead, and mummified. Data collected at the piglet level included sex, individual piglet weight at processing (Ind BWT; used as a proxy for birth weight), and the level of exposure to IUGR.

The level of IUGR a piglet was exposed to was determined by the shape of the head, using visual scores based on head morphology [34] and three criteria that characterise growth-restricted piglets [35], i.e. (1) steep, dolphin-like forehead; (2) bulging eyes; and (3) wrinkles perpendicular to the mouth. Using these criteria, the following three-point piglet head-morphology score was used: 1 = normal head shape, no criteria met; 2 = moderate IUGR head morphology, one or two criteria met; and 3 = severe IUGR head morphology, all criteria met. These scores were then further condensed into either a normal head shape (score 1) or an IUGR head morphology (scores 2 and 3 combined), which translated into the following traits: (1) at the piglet level, whether the piglet was born with a normal head shape or an IUGR head morphology (HEAD CLASS); and (2) at the sow level, the number of piglets with a HEAD CLASS of 1, which resulted in the proportion of piglets in a litter with an IUGR head morphology (PROP IUGR). For each litter, the average birth weight (AVE BWT), the standard deviation of birth weight (SD BWT), and the proportion of the litter (born alive and born dead) that survived to processing (18–24 h after birth; PROP SURV) were calculated.

### Statistical analyses

All genetic parameters, predicted means for the fixed effects, and covariance parameters for covariates at both the piglet and sow levels were obtained using mixed linear models in ASReml [44]. Traits analyzed included the binary trait HEAD CLASS at the piglet level, which was not transformed before analysis and although there are statistically more rigorous mathematical algorithms based on assumptions of ordered categories and concepts of thresholds [45–47], the general linear model has been shown to be an appropriate approximation [48].

Traits of interest at the piglet level were HEAD CLASS [normal (0) or IUGR (1)] and birth weight (Ind BWT). A bivariate mixed linear model was used that included fixed effects for sow parity (1–6 +), piglet sex [male (M) or female (F)], presence of mummified piglets within the litter (Yes/No), month of birth, and the covariates of litter size and gestation length. Random effects were the direct additive genetic effect, the permanent environmental effect of the dam, and the residual.

Traits of interest at the sow level were PROP IUGR, AVE BWT, SD WBT, LITTER SIZE, PROP SURV, and GL. A 6-variate mixed linear model was used with fixed effects for sow parity, farrowing month, and the covariate of gestation length. Random effects were the additive genetic effect (variance *V*_*g*_), permanent environmental effect (variance *V*_*pe*_), and the residual (variance *V*_*r*_) of the sow. Repeatability was calculated as: *P* = (*V*_*g*_ + *V*_*pe*_/*V*_*p*_), where *V*_*p*_ is the phenotypic variation of the trait. It should be noted that piglet genotype (L × LW or L × WD) was initially included in the analysis but since it was not significant (P > 0.1), it was removed from the model.

The pedigree structure that was used for the models at the piglet and sow levels is in Table 1. It should be noted that the pedigree structure did not allow separation of maternal additive effects, maternal permanent environmental effects and temporary environmental effects for any of the piglet level traits.

**Table 1:** Pedigree structure for datasets at the piglet and sow levels

Dataset	Records	Sires^a^	Dams^a^	Total pedigree size
Piglet level	21,159	58	861	23,436
Sow level	1575	133	438	2104

^a^Number of sires or dams with offspring with records

## Results

Summarized data for the piglet and sow levels are in Table 2. For the 1575 farrowings that were included in the study, 330 sows had one record, 345 two records and 185 three records. Parity ranged from 1 to 9, with 420, 322, 252, 223, 172, 97, 65, 20, and 4 litters, respectively. At the piglet level, more piglets were born with a normal HEAD-CLASS score (score 0; n = 17,604) than with an IUGR HEAD score (score 1; n = 3387), and Ind BWT ranged from 178 to 2960 g. At the litter level, there were 475 l with no piglets showing IUGR head morphology (PROP IUGR = 0). Ave BWT of a litter ranged from 820 to 2740 g and the SD BWT ranged from 8.5 to 811.8 g. LITTER SIZE ranged from 1 to 29 piglets. The within-litter proportion of piglets surviving to processing (18–24 h; PROP SURV) ranged from 0.086 to 1 and GL ranged from 106 to 120 d.

**Table 2:** Data summary at the piglet and sow levels

Trait	N	Mean	SD
*Piglet level*			
HEAD CLASS (0/1)	20,991	0.1614	0.3679
Ind BWT (g)	21,147	1453	370.3
*Sow level*			
PROP IUGR	1575	0.1445	0.1528
Ave BWT (g)	1575	1493	261.5
SD BWT (g)	1575	278.2	89.32
LITTER SIZE	1575	13.45	3.684
PROP SURV	1575	0.9013	0.1287
GL (days)	1575	114.9	1.260

Traits at the piglet level: HEAD CLASS = head morphology (normal or IUGR) and Ind BWT = individual birth weight

Traits at the sow level: PROP IUGR = proportion of piglets with an IUGR head morphology within a litter, AVE BWT = average birth weight within a litter, SD BWT = within-litter standard deviation in birth weight, litter size, PROP SURV = proportion of a litter surviving to processing, and GL = gestation length

*SD* standard deviation

For all traits, month of birth was a significant source of variation but these results are not presented since month of birth and any differences in management are not repeatable and are therefore of limited interest.

### Fixed effects for traits at the piglet level

Male piglets had a lower (more normal) HEAD CLASS score and were heavier at birth than female piglets (Table 3). Piglets from primiparous sows had a higher abnormal HEAD CLASS score than piglets from later parity sows (Table 4). Piglets from second parity sows had the lowest HEAD CLASS score, which then increased with successive parities. Ind BWT was lowest for primiparous sows, highest for second and third parity sows, and then decreased with successive parities (Table 4).

**Table 3:** Effects of piglet sex and presence of at least one mummified litter mate on traits at the piglet level

Piglet sex	Male	Female	P value
HEAD CLASS	0.168 ± 0.009	0.194 ± 0.009	0.005
Ind BWT (g)	1452 ± 16	1411 ± 16	< 0.001

Mummified litter mate	No	Yes	P value
HEAD CLASS	0.168 ± 0.009	0.193 ± 0.0111	0.040
Ind BWT (g)	1452 ± 15	1411 ± 17	0.007

HEAD CLASS = head morphology (score = 0 normal to 1 IUGR head morphology)

Ind BWT = individual birth weight

All standard errors (± SEM) are from a 2-variate model

**Table 4:** Effect of sow parity on traits at the piglet level

Sow parity	1	2	3	4	5	6 +
HEAD CLASS	0.214 ± 0.011^a^	0.148 ± 0.011^c^	0.158 ± 0.011^c^	0.180 ± 0.011^b^	0.184 ± 0.012^b^	0.200 ± 0.013^ab^
Ind BWT (g)	1306 ± 18^c^	1473 ± 17^a^	1476 ± 17^a^	1456 ± 17^a^	1449 ± 18^a^	1427 ± 19^b^

HEAD CLASS = head morphology (score = 0 normal to 1 IUGR head morphology)

Ind BWT = individual birth weight

All standard errors (± SEM) are from bivariate model analyses of HEAD CLASS and Ind BWT

Within a line in the table, mean values that share the same character in superscript (a, b or c) do not differ significantly (*P* > 0.05)

Piglets from larger litters had a higher abnormal HEAD CLASS score (regression coefficient ± SEM, 0.017 ± 0.001, *P* < 0.001) and lower Ind BWT (regression coefficient ± SEM, − 40.26 ± 1.03, *P* < 0.001) than piglets from smaller litters. Piglets from a litter that had one (or more) mummified piglets had a higher abnormal HEAD CLASS score and lower Ind BWT than piglets from litters without mummified piglets (Table 3).

### Heritabilities for traits at the piglet level

The bivariate genetic analysis for traits at the piglet level was conducted on the full dataset of 21,159 records, of which, 20,991 piglets had both HEAD CLASS and Ind BWT records. For HEAD CLASS and Ind BWT, the permanent environmental effect of litter accounted for 5 and 15% of the phenotypic variance, respectively, while direct additive genetic effects for 5 and 20% of the phenotypic variance, respectively (Table 5). The residual accounted for 90 and 65% of the phenotypic variance, respectively.

**Table 5:** Estimates of phenotypic variance (*V*_*p*_), residual variance (*V*_*r*_), direct additive genetic variance (*V*_*g*_), maternal genetic variance (*V*_*m*_), common environmental effects within litter variance (*V*_*e*_), of direct and maternal heritability (in italics on diagonal), and of genetic correlations (below diagonal) for traits at the piglet level

	HEAD CLASS	Ind BWT
*V*_*p*_	0.131 ± 0.001	119,480 ± 2290
*V*_*r*_	0.121 ± 0.001	86,549 ± 1761
*V*_*g*_	0.002 ± 0.002	8822 ± 2970
*V*_*m*_	0.004 ± 0.001	12,313 ± 3420
*V*_*e*_	0.004 ± 0.001	11,793 ± 2614
Direct heritability for HEAD CLASS	*0*.*01* ± 0.007	
Direct heritability for Ind BWT	− 0.90 ± 0.099	*0*.*07* ± 0.024
Maternal heritability for HEAD CLASS	*0*.*03* ± 0.009	
Maternal heritability for Ind BWT	− 0.85 ± 0.077	*0*.*10* ± 0.028
Direct evolvability	7.68%	0.42%

HEAD CLASS = head morphology (score = 0 normal to 1 IUGR head morphology)

Ind BWT = individual birth weight

All standard errors (± SEM) are from a 2-variate model

Estimates of direct heritability for HEAD CLASS score and Ind BWT were low, i.e. 0.01 ± 0.001 and 0.07 ± 0.023, respectively (Table 5). The genetic correlation between these traits was high and negative, i.e. 0.93 ± 0.05. HEAD CLASS had a low *I*_*A*_ of 7.7% and Ind BWT had a very low *I*_*A*_ of 0.4%. The maternal heritability for HEAD CLASS was also low at 0.03 ± 0.009 but was slightly higher for Ind BWT at 0.11 ± 0.028.

### Fixed effects for traits at the sow level

Primiparous sows had the highest proportion of piglets with an IUGR head morphology (IUGR PROP), the lowest average birth weight (Ave BWT), the smallest variation in birth weight (SD BWT), and were among the highest for survival to processing (SURV PROP; Table 6). Second and third parity sows had the lowest IUGR PROP, which then increased as parity increased, and the highest Ave BWT, which then decreased as parity increased. The within-litter variability in birth weight (SD BWT) increased as parity increased. LITTER SIZE peaked at parities 4 and 5 and then decreased in older sows. The proportion of piglets surviving to processing (SURV PROP) was lowest in the oldest sows. Sow parity had no effect on gestation length (GL).

**Table 6:** Effect of sow parity on traits at the sow level

Parity	1	2	3	4	5	6 +
PROP IUGR	0.19 ± 0.01^c^	0.12 ± 0.01^a^	0.12 ± 0.01^a^	0.16 ± 0.01^b^	0.16 ± 0.01^b^	0.16 ± 0.01^b^
Ave BWT	1371 ± 25^a^	1552 ± 24^a^	1552 ± 24^a^	1501 ± 24^b^	1522 ± 25^ab^	1508 ± 24^ab^
SD BWT	241 ± 6^a^	271 ± 6^c^	281 ± 7^c^	303 ± 7^b^	308 ± 8^bc^	317 ± 7^bc^
LITTER SIZE	13.5 ± 0.27^ab^	13.4 ± 0.27^ab^	13.3 ± 0.29^ab^	14.2 ± 0.29^c^	13.9 ± 0.32^bc^	13.1 ± 0.31^a^
PROP SURV	0.90 ± 0.01^b^	0.90 ± 0.01^b^	0.91 ± 0.01^b^	0.89 ± 0.01^ab^	0.90 ± 0.01^ab^	0.88 ± 0.01^a^
GL	114.9 ± 0.11^a^	115.0 ± 0.10^a^	115.0 ± 0.11^a^	115.0 ± 0.11^a^	114.9 ± 0.12^a^	115.1 ± 0.11^a^

PROP IUGR = proportion of piglets with an IUGR head morphology within a litter

AVE BWT = average birth weight within a litter

SD BWT = within-litter standard deviation in birth weight

LITTER SIZE = litter size

PROP SURV = proportion of a litter surviving to processing

GL = gestation length

All standard errors (± SEM) are from a 6-variate model

Within a line in the table, mean values that share a common character in superscript (a, b and c) do not differ significantly (*P* > 0.05)

### Heritabilities for traits at the sow level

Genetic analysis for traits at the sow level was conducted on the full dataset of 1575 records, with all sows having a complete set of records. For all traits, estimates of variance of the animal permanent environmental effect, direct additive genetic effects, and residual variance ranged from 4 to 14%, 6 to 33% and 59 to 84% of the phenotypic variance, respectively. Heritabilities for traits at the sow level ranged from low to moderate values (Table 7): PROP IUGR, 0.20 ± 0.05; Ave BWT, 0.33 ± 0.07; SD BWT, 0.12 ± 0.04; LITTER SIZE, 0.11 ± 0.05; PROP SURV, 0.04 ± 0.03; and GL, 0.20 ± 0.05. Estimates of the genetic correlation of PROP IUGR with the other traits were all moderate to high and negative (− 0.29 to − 0.90), with the exception of the correlation with LITTER SIZE, which was moderate and positive (0.46; Table 7). This shows that as PROP IUGR increases, Ave BWT and PROP SURV decrease. However, it should be noted that, in this dataset, not all estimated genetic correlations were significantly different from zero. PROP IUGR had a moderate *I*_*A*_ of 21.6% but was about 1% or less for all other traits. All repeatabilities were higher than the estimated heritabilities (Table 7), except for Ave BWT for which they were identical, which indicate that some non-genetic-selection gains can be made for those traits.

**Table 7:** Estimates of phenotypic variance (*V*_*p*_), residual variance (*V*_*r*_), genetic variance (*V*_*g*_), permanent environmental effect of the animal variance (*V*_*pe*_), and of heritabilities (in italics on diagonal) and phenotypic (above the diagonal) and genetic correlations (below diagonal) for traits at the sow level

	PROP IUGR	Ave BWT	SD BWT	LITTER SIZE	PROP SURV	GL
*V*_*p*_	0.022 ± 0.0009	64,673 ± 2786	7325 ± 273.16	13.11 ± 0.495	0.016 ± 0.0006	1.533 ± 0.0595
*V*_*r*_	0.016 ± 0.0008	38,710 ± 2008	6107 ± 308.73	9.82 ± 0.503	0.014 ± 0.0007	1.146 ± 0.0588
*V*_*g*_	0.005 ± 0.0013	21,303 ± 5084	864 ± 341.57	1.42 ± 0.611	0.0007 ± 0.0005	0.311 ± 0.0864
*V*_*pe*_	0.002 ± 0.001	4661 ± 4124	354 ± 361.40	1.87 ± 0.671	0.002 ± 0.0008	0.076 ± 0.0802
PROP IUGR	*0*.*20* ± 0.05	− 0.68 ± 0.01	0.27 ± 0.02	0.38 ± 0.02	− 0.20 ± 0.02	− 0.12 ± 0.03
Ave BWT (g)	− 0.90 ± 0.06	*0*.*33* ± 0.07	− 0.07 ± 0.03	− 0.60 ± 0.02	0.27 ± 0.02	0.14 ± 0.03
SD BWT (g)	− 0.29 ± 0.24	0.60 ± 0.18	*0*.*12* ± 0.04	0.19 ± 0.03	− 0.12 ± 0.03	− 0.05 ± 0.03
LITTER SIZE	0.46 ± 0.20	− 0.59 ± 0.15	− 0.52 ± 0.29	*0*.*11* ± 0.05	− 0.14 ± 0.02	− 0.19 ± 0.03
PROP SURV	− 0.80 ± 0.32	0.84 ± 0.29	0.53 ± 0.41	− 0.62 ± 0.36	*0*.*04* ± 0.03	0.06 ± 0.03
GL (days)	− 0.36 ± 0.19	0.21 ± 0.18	− 0.18 ± 0.23	− 0.34 ± 0.23	0.58 ± 0.32	*0*.*20* ± 0.05
Evolvability (*I*_*A*_)	23.95%	0.96%	1.12%	0.78%	0.09%	0.002%
Repeatability	0.29 ± 0.03	0.33 ± 0.07	0.16 ± 0.04	0.24 ± 0.03	0.13 ± 0.04	0.25 ± 0.04

All standard errors (± SEM) are from a 6-variate model

PROP IUGR = proportion of HEAD CLASS 1 piglets within a litter

AVE BWT = average birth weight within a litter

SD BWT = within litter standard deviation in birth weight

PROP SURV = proportion of a litter surviving to processing

GL = gestation length

## Discussion

Changes in piglet head morphology are known to be a phenotypic indicator of IUGR [IUGR; 34, 35], with IUGR challenge resulting in a specific head morphology that is easy to characterise on farm and under commercial conditions. Our results indicate that genetic gains would be minor when selection is against IUGR at the piglet level; however, selection against the within-litter proportion of IUGR at the sow level would yield better results and may be a suitable trait for an indirect measure of uterine capacity.

At the piglet level, gestation length had a slight effect on IUGR status and birth weight, with a shorter gestation length associated with a higher HEAD CLASS score, i.e. a more IUGR like head morphology and a lower birth weight. In general, shorter gestation lengths are associated with lower birth weights and more intrapartum deaths [49], while longer gestation lengths are often associated with higher birth weights [50]. The association between gestation length and HEAD CLASS score may be due to the negative genetic relationship between gestation length and litter size at the maternal level, with larger litters being associated with shorter gestation lengths, and the negative genetic relationship between litter size and proportion of IUGR in a litter, with larger litters being associated with a higher proportion of IUGR head morphology and lower birth weight piglets. It should be noted that not all genetic correlation estimates were significantly different from zero (with high standard errors). Studies with more animals and different populations are necessary to elucidate furthermore these relationships and their impact on IUGR incidence. Our heritability estimate for gestation length and the associated genetic correlations fit squarely with values in the literature, i.e. heritabilities for gestation length ranging from 0.14 to 0.30 [51–53], with a negative genetic correlation with litter size [51] and a positive genetic correlation with average birth weight [53].

Our findings agree with those from other studies in which first parity sows produce piglets with lower average birth weight than older parities. Sows are not physically mature during their first gestation and do not reach maturity until approximately 18 months of age [54], although some studies have shown that body growth continues past the 6th parity [55]. Because of this, young sows are ‘selfish’ in terms of nutrient partitioning, keeping more of their available nutrients for somatic body growth rather than offspring growth. This results in more nutritional retardation and thus lower birth weights and a higher proportion of piglets with an IUGR head morphology. In addition, birth weight varies less within first parity litters because more of these litters are uniformly small. It is interesting to note that, in our study, second parity sows do not follow the biological logic in energy partitioning, since they are expected to still have significant somatic growth and thus to be intermediate between first and third parity sows.

Litter size is a significant predictor of piglet IUGR head morphology. Recent selection pressure for increased litter size at birth has resulted in more piglets being born with a low birth weight [12, 13, 56, 57], lower physiological maturity at birth [58], and higher rate of intrauterine growth retardation [14]. The estimated heritability for litter size, in our study, is similar to those reported for other polytocous species [1, 3, 4, 59, 60], and typical for a composite reproductive trait. We also found that litter size and average birth weight at the sow level were strongly negatively genetically correlated and acted antagonistically on mother and offspring, which is in agreement with other studies [1, 3, 4, 60]. This antagonistic relationship, with a presumed negative genetic correlation between number and size of offspring is thought to constrain the evolution of litter size and average birth weight [2].

In theory, genetic correlations between life history traits are expected to be negative [61] and our estimate of the genetic correlation between litter size and average birth weight was high and negative. An increase in average birth weight has been linked to a reduced risk of mortality [62], which explains our high and positive genetic correlation estimate between average birth weight and within-litter proportion of piglets surviving till processing. The within-litter standard deviation in birth weight has also been linked to a greater odds-ratio for mortality of piglets [62], with this within-litter weight distribution already established by the end of the embryonic stage of gestation [63]. In our study, litter size was highly negatively genetically correlated with the proportion of piglets surviving till processing (18–24 h after birth), negatively genetically correlated with average birth weight, and positively genetically correlated with the within-litter proportion of IUGR piglets, which suggests that bigger the litter size, the lower the average birth weight, the greater the proportion of IUGR piglets, and the lower the proportion of piglets surviving until 24 h after birth. Of particular interest is the highly negative genetic correlation between the proportion of IUGR-affected piglets and the proportion of piglets surviving until processing, which indicates that selection against the within-litter proportion of IUGR-affected piglets would result in an associated increase in the within-litter proportion of piglets surviving until 24 h of age.

Uterine capacity can be defined as the number of conceptuses a uterus can successfully carry to term [64]. In general, the number of living conceptuses that the uterus is capable of supporting is larger in early gestation than later [38], while in later gestation intrauterine competition for the establishment of adequate surface area for nutrition exchange between foetal and maternal circulations may act to limit litter size [14]. However, it is often difficult to measure uterine capacity directly on farm and most selection programmes use indirect measures of uterine capacity, such as increasing litter size, selection on the number of live born piglets at day 5 [65] or on litter weight [66].

In our study, phenotypes were collected on the piglets and, thus, the underlying mechanism resulting in reduced incidences of IUGR is unclear since measures to characterise uterine morphology and physiology were not available. Selection for reduced rates of IUGR may result in improving the development of the folded placental-epithelial/maternal-epithelial bilayer and fold depth, i.e. the maternal foetal interactive surface [38], or of a more efficient placenta. However, a comparison of placental efficiency (the grams of foetus that could be supported by the grams of placenta) between Meishan and Yorkshire breeds highlighted that the greater placental efficiency of Meishan sows was a result of smaller conceptuses that contained fewer cells, compared with Yorkshire conceptuses regardless of uterine environment [67]. In addition, although selection for placental efficiency may result in greater litter size without decreasing piglet viability [68], the resulting piglets had a 20% lower birth weight than those of control lines. An alternative effect of selection for lower rates of IUGR may be more evenly spaced embryos. Lents et al. [69] reported negative relationships of ovulation rate with embryonic spacing and with empty space around the embryonic-placental unit, which both indicate crowded uterine conditions. Other studies have shown that mouse lines that lack lysophosphatidic acid, a phospholipid involved in uterine peristaltic movements that facilitate embryo separation [69], have blastocysts that remain clustered in the vicinity of the cervix, whereas non-deficient lines have blastocysts that are evenly distributed along the uterine horns [70].

Studies that specifically investigated different aspects of uterine capacity reported that genetic selection of gilts for high uterine capacity led to greater litter size and overall litter weight, but not to a greater average birth weight [71], and that selection for increased placental efficiency did not increase litter size [72]. Lents et al. [73] investigated the phenotypic differences of the reproductive tract in prepubertal gilts using transrectal ultrasound and found that, overall, selection for increased uterine capacity resulted in larger ovaries and uterine horn diameter but that there was no discernible difference in these traits between gilts selected for increased uterine capacity and control gilts at 130, 150 or 170 days, which suggests that uterine capacity is difficult to select for prior to first breeding.

Attempts to identify candidate genes [74] and chromosomal regions that harbour quantitative trait loci [75] found no definitive associations between major genes or markers for reproductive traits, although more recent studies have since identified some potential genetic markers for various reproductive traits average birth interval [76], teat number [77], e.g. age at puberty and reproductive longevity [78].

Another approach to estimate uterine capacity is to use the unilateral hysterectomy-ovariectomy (UHO) model. Christenson et al. [79] concluded that UHO gilts that carried the largest litters had greater uterine capacity. Regardless of the mechanism underlying selection for increased uterine capacity, this variability indicates that there is additive genetic variance in this trait.

In our study, the heritability of IUGR head morphology at the piglet level was low, i.e. 0.01. The proportion of maternal variance was also low, i.e. 0.03. In contrast, the heritability for birth weight was higher at 0.10, while the proportion of maternal variance was 0.14. However, it should be noted that, in this dataset, the direct heritability of the continuous birth weight trait was low compared with other studies [80, 81]. Sire effects are also known to affect piglet birth weight [82], but we did not find any significant effect of the piglet genotype (L × LW or L × WD) and, therefore, removed it from the model.

Binary traits tend to have a lower heritability than continuous traits by virtue of the binary scale and its lack of intermediate values, with the bias and precision of the estimation methods, depending on the incidence of the phenotype under study [83]. The conventional solution to binary traits is to treat the binary trait as if it was continuous, quantify the heritability and adjust the estimates based on the prevalence of the trait in the population. In our study, since the direct heritability for traits at the piglet level was sufficiently low, no further adjustments were conducted and an alternative trait (at the sow level) was investigated.

In general, traits with the strongest influence on survival have the lowest heritabilities [84–86]. There are two possible reasons for this: (1) fitness traits limit the evolutionary potential by exhausting the genetic variation for traits in direct proportion to their effect on fitness [85, 87]; or (2) fitness traits have greater residual variance [40, 42, 86, 88]. In our study, the residual variance of the IUGR head morphology trait at the piglet level was high, i.e. 90% of the phenotypic variance, compared to the 5% attributable to genetics, which suggests the second explanation may be more appropriate [89].

If offspring fitness depends on the piglet’s IUGR head morphology and associated effects, piglet head morphology is expected to have a higher evolvability than other morphological traits. However, an evolvability of ~ 8% was found for IUGR head morphology, which indicates that the potential phenotypic variation for this trait is small. In addition, the direct narrow-sense heritability was low at 0.01 due to the high residual variance and, thus, direct selection against IUGR head morphology at the piglet level would be slow. If selection against intrauterine growth retardation at the piglet level is not suitable/appropriate, is selection at the sow level a better prospect? Heritability of the proportion of IUGR-affected piglets in a litter was approximately 0.20. The variance percentage for the permanent environmental effect of the sow was low at 9%, while the residual variance accounted for 72% of the phenotypic variance. The evolvability for the within-litter IUGR proportion was approximately 22%, while the narrow-sense heritability was 0.20. The repeatability of the proportion of IUGR-affected piglets in a litter was 0.29 ± 0.03, which suggests that, although most of the improvement may be achieved via genetic selection some improvement can be obtained via the maternal environment.

Repeatability is the proportion of total variance in multiple measurements that is due to individual differences [90] and is useful for quantifying the extent to which an individual’s performance remains consistent over time [91–93]. The generally low repeatability of the traits analysed in this study may indicate practical problems that are associated with trait measurement or that the time-frame for measurement was not relevant [94]. This may be true for young sows, with primiparous sows having the highest proportion of IUGR-affected piglets and the lowest average birth weight compared with older sows, which is most likely due to the biology behind energy partitioning. Thus, these findings suggest that these traits should be treated as different traits in primiparous and older animals. However, the lower repeatabilities may also show a lack of independence for successive measurements [90] since, at each successive farrowing, the traits are context-dependent on litter size. The number of conceptuses will differ at each service for many reasons, i.e. poor/good sow body condition at service, poor quality semen sample, or reduced quality of available oocytes in older parity sows.

In this study, we show that selection against intrauterine growth retardation and an associated increase in the proportion of piglets surviving until 24 h, can be effective. In addition, selection for component traits of female reproduction/fertility does not appear to negatively affect boar semen quality [95], which means that the within-litter proportion of IUGR-affected piglets trait has potential application in the field. Its measurement is based on a visual score [34, 35], which is easily collected under commercial conditions. This indicates that selection against the within-litter proportion of IUGR-affected piglets, with the corresponding genetic gains in associated traits, is a suitable trait for use in commercial breeding programs.

In conclusion, selection against intrauterine growth retardation is possible at the maternal level, through the within-litter proportion of IUGR-affected piglets trait. Inclusion of litter size in addition to the within-litter proportion of IUGR-affected piglets in the breeding programme should maintain current litter sizes. An important and beneficial consequence of selection on low levels of within-litter proportions of IUGR-affected piglets would be a corresponding increase in the survival of piglets to 24 h of age.
